# Modified Aarhus Composite Biomarker Score as a New Risk-Stratification Tool in Metastatic Colorectal Cancer

**DOI:** 10.3390/diagnostics16060863

**Published:** 2026-03-13

**Authors:** Nagihan Kolkıran, Atike Pınar Erdoğan, Mustafa Şahbazlar, Ferhat Ekinci

**Affiliations:** Department of Medical Oncology, Faculty of Medicine, Manisa Celal Bayar University, Manisa 45030, Turkey; atike.erdogan@cbu.edu.tr (A.P.E.); mustafa.sahbazlar@cbu.edu.tr (M.Ş.); ekinci.ferhat@cbu.edu.tr (F.E.)

**Keywords:** metastatic colorectal cancer, modified Aarhus composite biomarker score, overall survival, prognostic tool

## Abstract

**Background/Objectives**: Systemic inflammatory markers are increasingly recognized as prognostic indicators in metastatic colorectal cancer (mCRC), demonstrating significant associations with survival outcomes. The aim of this study was to evaluate the prognostic value of the Aarhus composite biomarker score (ACBS) in patients with metastatic colorectal cancer and to introduce the modified ACBS as a laboratory-based prognostic tool in mCRC. **Methods**: The Aarhus Composite Biomarker Score was calculated using serum albumin, C-reactive protein (CRP), neutrophil count, lymphocyte count, and hemoglobin levels. The modified Aarhus Composite Biomarker Score-1 (mACBS-1) stratified patients into three prognostic groups: favorable, intermediate, and poor risk. The simplified modified Aarhus Composite Biomarker Score-2 (mACBS-2) categorized patients into two prognostic groups (low vs. high risk). Survival analyses were performed using the Kaplan–Meier method, and prognostic factors were evaluated using Cox regression analysis. **Results**: The median overall survival (OS) was 35 months (95% CI: 29.38–40.62). Stratification by mACBS-1 revealed median OS values of 47, 30, and 14 months for favorable-, intermediate-, and poor-risk groups, respectively (*p* = 0.002). Similarly, mACBS-2 distinguished two prognostic groups, with median OS of 47 months in the favorable-risk group and 30 months in the poor-risk group (*p* = 0.001). In multivariable analysis, ACBS remained an independent predictor of overall survival, with three abnormal biomarkers conferring a significantly increased mortality risk (HR 4.61, 95% CI 2.17–9.82, *p* < 0.001). Similarly, poor-risk classification by mACBS-1 (HR 3.36, 95% CI 1.58–7.12, *p* = 0.002) and mACBS-2 (HR 2.05, 95% CI 1.29–3.26, *p* = 0.002) was independently associated with worse survival. **Conclusions**: The ACBS and its modified versions (mACBS-1 and mACBS-2) are simple, laboratory-based prognostic tools with independent predictive value for survival in metastatic colorectal cancer. Its clinical use may support improved risk stratification and individualized patient management.

## 1. Introduction

Colorectal cancer (CRC) represents a major global health problem, remaining among the most frequently diagnosed malignancies and leading causes of cancer-related death worldwide [1]. While tumorigenesis in CRC is traditionally attributed to genetic and epigenetic alterations, increasing evidence indicates that host-related factors, particularly inflammation, play a critical role in disease initiation and progression [2]. Chronic inflammatory processes contribute to colorectal cancer development by shaping a tumor- permissive microenvironment, characterized by enhanced vascular formation, altered immune surveillance, and increased metastatic potential [3]. Experimental and clinical studies have demonstrated that inflammation-associated signaling cascades, including pathways involving NF-κB and STAT3, act as key molecular mediators linking inflammatory stimuli to tumor growth and dissemination [4].

From a clinical perspective, systemic inflammation has been increasingly recognized as a determinant of disease aggressiveness and patient outcomes in colorectal cancer [5]. In this context, readily accessible laboratory markers reflecting inflammatory status, such as CRP and immune cell-based indices, have attracted significant attention as potential prognostic tools [6]. Elevated inflammatory markers have been associated with advanced tumor stage, impaired treatment response, and reduced survival in colorectal cancer [6]. In addition to its direct effects on tumor biology, systemic inflammation contributes to cancer-associated cachexia, a complex metabolic syndrome that adversely affects physical function, treatment tolerance, and overall prognosis [7].

Within this inflammatory framework, individual inflammatory and nutritional biomarkers have demonstrated independent prognostic relevance in metastatic colorectal cancer. Elevated CRP levels, indicative of persistent systemic inflammation, have consistently been associated with poor overall survival and disease progression in mCRC [8]. Serum albumin, influenced by nutritional status and systemic inflammation, has emerged as a robust prognostic marker, with hypoalbuminemia correlating with adverse survival outcomes in colorectal cancer [9]. Hemoglobin-related measures, such as the hemoglobin-to-red cell distribution width ratio, have demonstrated independent prognostic significance for overall survival in metastatic colorectal cancer, reflecting the combined impact of anemia and systemic inflammation [10].

Consequently, recent research has focused on composite biomarkers that integrate multiple laboratory parameters to enhance prognostic discrimination. Several ratio- or formula-based indices have been explored in colorectal cancer. For example, the lymphocyte-to-C-reactive protein ratio (LCR) has been proposed as a surrogate marker of systemic inflammatory burden and has demonstrated favorable performance in predicting outcomes in colorectal cancer [11,12]. Similarly, indices derived from peripheral blood counts, including the systemic immune-inflammation index (SII), which is calculated using neutrophil, platelet, and lymphocyte counts, have been associated with survival in patients with metastatic colorectal cancer [13].

In contrast to ratio- or formula-based indices, the Aarhus Composite Biomarker Score (ACBS) is constructed using predefined abnormal thresholds of individual laboratory parameters—hemoglobin, albumin, CRP, neutrophil count, and lymphocyte count—rather than calculated ratios or mathematical combinations. ACBS has shown prognostic value in other malignancies, such as bone sarcomas and non-small cell lung cancer [14,15]. However, its prognostic relevance in metastatic colorectal cancer has not yet been sufficiently investigated. Therefore, this study aimed to evaluate the prognostic performance of ACBS and to develop a modified ACBS to improve risk stratification for overall survival in this patient population.

## 2. Materials and Methods

This retrospective cohort study included 179 patients with metastatic colorectal cancer treated at the Medical Oncology Department of Manisa Celal Bayar University Faculty of Medicine between 2010 and 2025. Inclusion criteria were age ≥ 18 years, histopathologically confirmed colorectal adenocarcinoma, and either metastatic progression following prior treatment or synchronous metastatic disease at initial diagnosis. Exclusion criteria included age < 18 years and poor performance status precluding guideline-concordant systemic therapy. All clinicopathological and laboratory data were extracted from electronic medical records. Recorded variables include demographic characteristics, primary tumor location, metastatic presentation (recurrent versus de novo), sites of metastatic involvement, mutational status, Eastern Cooperative Oncology Group (ECOG) performance status, systemic chemotherapy regimens (including type and number of treatment lines), and baseline laboratory parameters obtained at the time of metastatic diagnosis including hemoglobin, lymphocyte count, neutrophil count, albumin, and CRP.

The Aarhus Composite Biomarker Score was calculated as previously described [14,15]. A score of 0 was assigned to patients who met all predefined biomarker thresholds: albumin ≥ 35 g/L, C-reactive protein < 8 mg/L, neutrophil count ≤ 7 × 10^9^/L, lymphocyte count ≤ 3.5 × 10^9^/L, and hemoglobin ≥ 7.3 mmol/L for women or ≥8.3 mmol/L for men. Scores of 1, 2, or 3 were assigned based on the presence of one, two, or three abnormal biomarker values, respectively, as defined by the predefined thresholds. All laboratory variables incorporated into the ACBS were collected at baseline, defined as the time of metastatic diagnosis and prior to the initiation of systemic therapy thus representing pretreatment status. Serial assessments and post-treatment changes in ACBS were not evaluated in this retrospective study.

In the present study, ACBS was further modified to develop a novel prognostic index, designated as mACBS-1. Patients were classified into three prognostic categories: favorable, intermediate, and poor risk. Favorable-risk patients fulfilled all predefined biomarker thresholds, whereas those with one or two abnormal values were categorized as intermediate risk and those with three or more abnormalities as poor risk. To enhance clinical applicability, a simplified two-tier classification system, termed mACBS-2, was subsequently developed. Patients with all normal biomarkers or only one abnormal value were classified as favorable risk, while those with two or more abnormal biomarker values were categorized as poor risk. The biomarker thresholds used to define abnormal values were identical to those in the original ACBS, and no ROC-based optimization of cut-off values was performed in this cohort.

Clinical outcomes were assessed based on radiographic response evaluation according to RECIST version 1.1 criteria and survival outcomes. Overall survival was defined as the time from the diagnosis of metastatic colorectal cancer to death from any cause or the date of last follow-up.

Statistical analyses were performed using IBM SPSS Statistics for Windows, version 27.0 (IBM Corp., Armonk, NY, USA). Descriptive statistics were used to summarize patient characteristics; categorical variables were presented as frequencies and percentages, while continuous variables were expressed as mean ± standard deviation, median, and range (minimum–maximum). Overall survival was estimated using the Kaplan–Meier method, and differences between survival curves were assessed using the log-rank test. Univariate and multivariate Cox proportional hazards regression analyses were conducted to identify independent prognostic factors associated with OS. The prognostic performance of ACBS and its modified versions was compared using Harrell’s C-index, time-dependent AUC calculated at 12, 36, and 60 months, and Akaike Information Criterion (AIC). A two-sided *p* value < 0.05 was considered statistically significant.

## 3. Results

### 3.1. Patient Characteristics

This retrospective cohort included 179 patients with metastatic colorectal cancer. The median age at diagnosis was 61.8 years (SD ± 12.8; range, 38–82 years), with a higher proportion of male patients (60.3%). Baseline demographic and clinicopathological characteristics are summarized in Table 1.

The primary tumor was most frequently located in the rectum (35.8%), followed by the sigmoid colon (30.7%) and the right colon (19.0%). At diagnosis, 64.2% of patients presented with de novo metastatic disease, while the remaining patients developed metastatic recurrence after prior treatment. The most common sites of metastasis were the liver (65.9%) and lungs (39.3%), with other distant extrahepatic or extrapulmonary sites observed in 49.2% of cases.

Mutational analysis showed RAS mutations in 63.1% of patients and BRAF V600E mutations in 5.6%. The distribution of mutations was also evaluated across ACBS, mACBS-1, and mACBS-2 categories. In the ACBS classification, KRAS mutations were observed in 64.5%, 61.1%, 66.0%, and 56.2% of patients in the normal, one abnormal, two abnormal, and three abnormal groups, respectively. BRAF mutations were detected in 11.3%, 1.9%, 4.3%, and 0.0% of patients in the corresponding groups, as shown in Supplementary Table S1. Similarly, in the mACBS-1 classification, KRAS mutations were present in 65.1%, 63.0%, and 56.2% of patients in the favorable, intermediate, and poor-risk groups, respectively, while BRAF mutations were observed in 11.1%, 3.0%, and 0.0% of patients (Supplementary Table S2). In the mACBS-2 classification, KRAS mutations were detected in 63.2% of patients in the normal/one abnormal group and 62.9% in the ≥2 abnormal group, whereas BRAF mutations were observed in 6.8% and 3.2% of patients, respectively (Supplementary Table S3). Overall, no statistically significant differences were observed in the distribution of KRAS or BRAF mutations across ACBS, mACBS-1, or mACBS-2 categories.

The distribution of systemic treatment regimens across ACBS and modified ACBS risk groups was also evaluated. Across all classifications, chemotherapy combined with anti-VEGF therapy represented the most frequently administered regimen, ranging from 68.1% to 87.5% across ACBS categories, 68.0% to 87.5% across mACBS-1 categories, and 74.2% to 74.4% across mACBS-2 categories. In contrast, chemotherapy combined with anti-EGFR therapy was administered in 12.5–31.9%, 19.0–32.0%, and 25.6–25.8% of patients across ACBS, mACBS-1, and mACBS-2 groups, respectively. No statistically significant differences were observed in treatment distribution across ACBS (*p* = 0.198), mACBS-1 (*p* = 0.082), or mACBS-2 (*p* = 1.000) risk groups (Supplementary Table S4).

### 3.2. Univariate and Multivariate Analyses of Overall Survival

Cox regression analyses were performed to identify prognostic factors associated with overall survival. Univariate Cox regression analysis demonstrated that several clinicopathological and biomarker-based variables, including metastatic disease at diagnosis, liver metastasis, and ACBS, were significantly associated with overall survival (Table 2). The presence of metastatic disease at diagnosis was associated with a markedly increased risk of mortality (HR = 2.161, 95% CI: 1.495–3.123; *p* < 0.001), while liver metastasis was similarly linked to poorer survival outcomes (HR = 1.822, 95% CI: 1.250–2.657; *p* = 0.002). In contrast, surgical resection of the primary tumor was associated with a significantly reduced risk of death (HR = 0.497, 95% CI: 0.319–0.773; *p* = 0.002).

Within the ACBS classification, patients with two abnormal biomarkers exhibited a more than two-fold increased risk of death compared with those with all normal biomarker values (HR = 2.326; 95% CI: 1.475–3.670; *p* < 0.001), whereas patients with three abnormal biomarkers demonstrated an even higher mortality risk (HR = 2.819; 95% CI: 1.494–5.319; *p* = 0.001).

Consistent with these findings, both modified scoring systems showed robust prognostic relevance. According to the mACBS-1 classification, patients with one or two abnormal biomarkers had an increased risk of death compared with the favorable-risk group (HR = 1.619; 95% CI: 1.099–2.387; *p* = 0.015), while those with three or more abnormal biomarkers exhibited a substantially higher mortality risk (HR = 2.776; 95% CI: 1.474–5.228; *p* = 0.002). In the simplified mACBS-2 model, poor-risk patients (two or more abnormal biomarkers) had a more than two-fold increased risk of death compared with favorable-risk patients (HR = 2.105; 95% CI: 1.469–3.017; *p* < 0.001).

Multivariate Cox regression analysis confirmed metastatic disease at diagnosis, bone metastasis, ACBS, mACBS-1, and mACBS-2 as independent prognostic factors for overall survival, as shown in Table 3. Metastatic disease at diagnosis was independently associated with an increased risk of mortality (HR, 1.75; 95% CI, 1.11–2.74; *p* = 0.015), as was the presence of bone metastasis (HR, 1.90; 95% CI, 1.08–3.34; *p* = 0.025). Within the ACBS classification, patients with two abnormal biomarkers had a significantly higher risk of death compared with those with all normal biomarker values (HR, 1.84; 95% CI, 1.03–3.29; *p* = 0.040), while patients with three abnormal biomarkers exhibited a markedly increased mortality risk (HR, 4.61; 95% CI, 2.17–9.82; *p* < 0.001). Consistently, the modified scoring systems retained independent prognostic significance. In the mACBS-1 model, poor-risk patients (three or more abnormal biomarkers) demonstrated a more than threefold increased risk of death compared with favorable-risk patients (HR, 3.36; 95% CI, 1.58–7.12; *p* = 0.002). Similarly, in the simplified mACBS-2 model, poor-risk patients had a significantly higher mortality risk than favorable-risk patients (HR, 2.05; 95% CI, 1.29–3.26; *p* = 0.002).

### 3.3. Survival Outcomes

**Figure 1:**
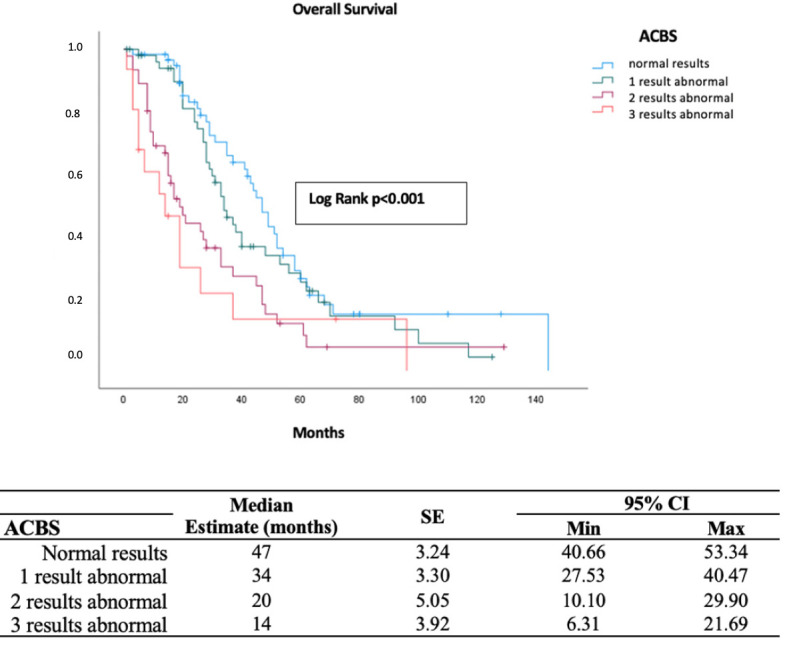
Kaplan–Meier analysis of overall survival stratified by the Aarhus composite biomarker score.

The cohort demonstrated a median OS of 35 months (95% CI: 29.38–40.62), with survival rates of 85.6% at 1 year, 47.9% at 3 years, 23.2% at 5 years, and 7.8% at 10 years. Survival analysis stratified by ACBS revealed a significant difference in OS among risk groups. Patients with all normal biomarker values exhibited the longest median OS of 47 months (95% CI: 40.66–53.34), whereas those with one, two, or three abnormal biomarkers demonstrated progressively shorter median OS values of 34, 20, and 14 months, respectively (*p* < 0.001; Figure 1).

**Figure 2:**
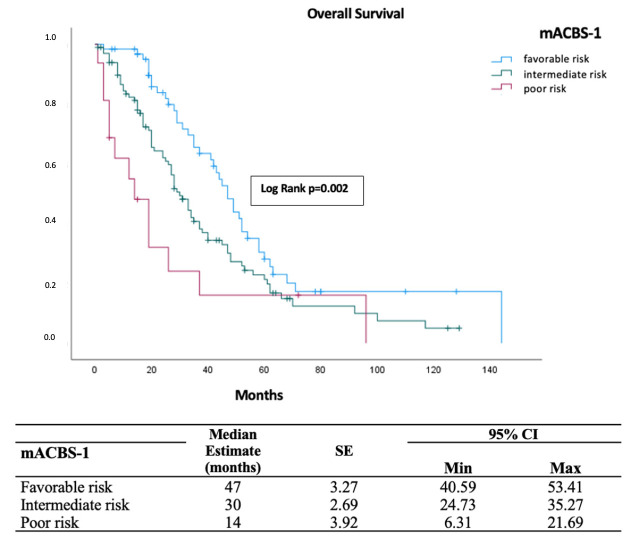
Kaplan–Meier analysis of overall survival stratified by the modified Aarhus composite biomarker score-1.

**Figure 3:**
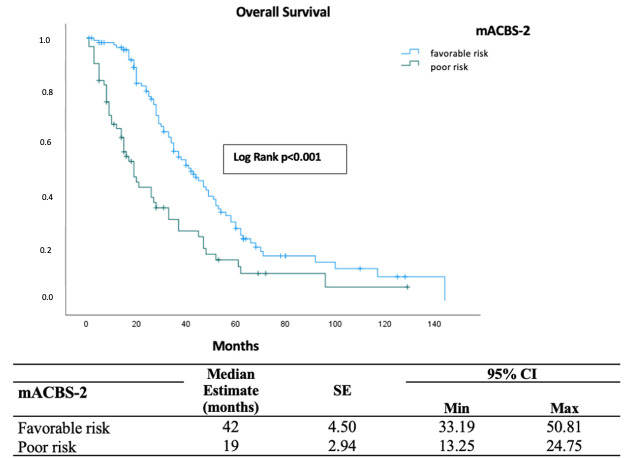
Kaplan–Meier analysis of overall survival stratified by the modified Aarhus composite biomarker score-2.

The mACBS-1 classification provided enhanced risk stratification by identifying three prognostic cohorts with significantly different survival outcomes (*p* = 0.002; Figure 2). Patients in the favorable-risk group showed superior survival, with a median OS of 47 months (95% CI: 40.59–53.41). In contrast, intermediate-risk patients had a median OS of 30 months (95% CI: 24.73–35.27), while the poor-risk group exhibited a markedly shorter median OS of 14 months (95% CI: 6.31–21.69). The mACBS-2 classification demonstrated comparable prognostic performance, with favorable-risk patients achieving a median OS of 42 months (95% CI: 33.19–50.81), compared with 19 months (95% CI: 13.25–24.75) in poor-risk patients (*p* < 0.001; Figure 3).

### 3.4. Comparison of Prognostic Performance Between ACBS and Modified ACBS Models

The original ACBS demonstrated the highest discriminatory capacity, with a Harrell’s C-index of 0.659. Time-dependent AUC values were 0.818, 0.674, and 0.580 at 12, 36, and 60 months, respectively (mean AUC 0.690). The mACBS-2 model showed slightly lower discriminatory performance (Harrell’s C-index 0.632; mean AUC 0.663) but yielded the lowest AIC value (1081.97), indicating improved model parsimony. In contrast, the mACBS-1 model demonstrated the lowest performance across discrimination metrics (Harrell’s C-index 0.620; mean AUC 0.642; AIC 1088.18) (Supplementary Table S5).

## 4. Discussion

Systemic inflammation is increasingly recognized as a central biological process driving cancer progression through its effects on tumor growth, metastatic dissemination, and immune suppression [2,16]. This interaction between tumor biology and host inflammatory response has prompted extensive investigation into inflammation-based prognostic biomarkers, particularly routinely available hematologic indices such as the neutrophil-to-lymphocyte ratio (NLR), platelet-to-lymphocyte ratio (PLR), and the systemic immune-inflammation index (SII), all of which have demonstrated prognostic relevance in colorectal cancer [4,17]. These markers reflect both tumor-induced inflammation and host immune dysregulation, providing clinically accessible tools for risk stratification.

Within this framework, prior studies have highlighted the prognostic importance of composite inflammatory indices in metastatic colorectal cancer. For example, integration of SII with tumor-infiltrating lymphocytes has been shown to improve prognostic discrimination, emphasizing the interplay between systemic inflammation and antitumor immunity [17]. Similarly, elevated pretreatment SII levels have been associated with inferior progression-free survival in patients receiving bevacizumab-based therapy, further supporting the clinical relevance of inflammation-derived biomarkers in treatment outcomes [18].

Beyond conventional inflammatory indices, several composite scoring systems have been developed to capture the multifaceted nature of systemic inflammation. The Systemic Inflammation Response Index (SIRI), calculated as neutrophil × monocytes/lymphocytes, has been reported as a significant predictor of metastatic potential and disease stage in colorectal cancer [19]. In parallel, the Glasgow Prognostic Score (GPS), which integrates CRP and albumin levels, has been widely used to assess inflammation-related survival risk across multiple malignancies, including colorectal cancer [20]. These composite models underscore the added value of combining multiple laboratory parameters over single biomarkers when evaluating cancer prognosis.

In this context, the Aarhus Composite Biomarker Score, incorporating CRP, albumin, neutrophil count, lymphocyte count, and hemoglobin, represents a comprehensive approach to assessing systemic inflammation and nutritional status. Previous investigations have validated the prognostic utility of ACBS in several malignancies. In localized bone sarcomas, ACBS was shown to predict disease-specific mortality independently of comorbidities and other clinical factors [14]. Subsequent studies in metastatic sarcoma further confirmed its reliability as a prognostic tool for disease-specific survival [21]. Moreover, ACBS has demonstrated robust prognostic performance in non-small cell lung cancer, where higher scores were consistently associated with poorer overall and progression-free survival [15]. Notably, ACBS outperformed established indices such as GPS and NLR in this setting, highlighting the benefit of integrating multiple inflammatory parameters into a single scoring system [15].

To our knowledge, this study represents the first dedicated and systematic evaluation of ACBS for survival prediction in patients with metastatic colorectal cancer. Our findings demonstrate that higher ACBSs are significantly associated with inferior overall survival in mCRC, reinforcing the pivotal role of systemic inflammation in disease progression. Unlike previous reports in other malignancies, we did not observe a statistically significant survival difference between patients with ACBSs of 0 and 1, which may be attributable to sample size limitations and cohort-specific characteristics.

Importantly, we extended the clinical applicability of ACBS by developing two modified classification systems, mACBS-1 and mACBS-2, designed to enhance risk stratification in metastatic colorectal cancer. Both modified models effectively stratified patients into distinct prognostic groups, with progressively worse survival outcomes observed in higher-risk categories. The simplified two-tier mACBS-2 model, in particular, may offer practical advantages in routine clinical settings by facilitating rapid risk assessment using readily available laboratory data. Although the original ACBS demonstrated the highest prognostic discrimination, the simplified mACBS-2 model showed the lowest AIC, suggesting that the two-tier model may offer practical advantages for clinical application. Importantly, the significant differences observed in log-rank tests and multivariable Cox regression analyses indicate that ACBS retains prognostic discrimination despite the partial convergence of Kaplan–Meier curves at later follow-up.

The consistent prognostic performance of ACBS and its modified versions in mCRC suggests that this scoring system reflects systemic host-related conditions, particularly inflammatory and nutritional status, that may be relevant across diverse oncologic contexts. Although the individual components of ACBS reflect systemic inflammatory and nutritional status that are biologically relevant beyond malignancy, the ACBS model itself has primarily been developed and applied in oncologic populations for prognostic assessment. In the present study, ACBS was evaluated exclusively as a prognostic risk stratification tool and was not intended to serve as a predictive biomarker for treatment response or treatment monitoring. All patients received standard-of-care systemic therapy independent of ACBS classification. Therefore, higher-risk categorization should be interpreted as reflecting increased baseline inflammatory and nutritional burden rather than reduced treatment eligibility or diminished therapeutic benefit.

Collectively, our results support two fundamental oncologic principles: first, that systemic inflammatory and nutritional biomarkers play a critical role in determining cancer prognosis; and second, that composite biomarker models such as ACBS can be successfully applied across different disease contexts. In addition, the distribution of major oncogenic mutations did not significantly differ across ACBS categories in our cohort. KRAS and BRAF mutation frequencies were comparable between risk groups, suggesting that the prognostic performance of ACBS appears to be largely independent of major genomic alterations.

The present study has several limitations. First, its retrospective design and relatively modest sample size may introduce potential selection bias and may limit the generalizability of the findings. Second, as a single-center analysis, the patient population may not fully represent the broader spectrum of metastatic colorectal cancer treated in different clinical settings. Another limitation of the present study is that ACBS was assessed exclusively at baseline, and serial measurements during the disease course were not available. Prospective investigations integrating longitudinal ACBS assessments may further clarify its potential utility as a dynamic treatment-monitoring biomarker.

Despite these limitations, several aspects support the scientific relevance of our results. The cohort represents a real-world population of patients with metastatic colorectal cancer, and the prognostic associations of ACBS and its modified versions remained consistent across both univariate and multivariate analyses. Moreover, the observed effect sizes were clinically meaningful, demonstrating clear survival differences between risk groups. Taken together, these findings suggest that ACBS and its modified versions may provide robust prognostic stratification despite the modest cohort size. Further validation in larger and independent cohorts will be important to confirm the generalizability of these findings.

In summary, the Aarhus Composite Biomarker Score and its modified versions, which integrate routinely available inflammatory and nutritional parameters, represent practical and non-invasive tools for prognostication in patients with metastatic colorectal cancer. By combining multiple laboratory-based biomarkers reflecting systemic inflammation, these models may improve risk stratification and assist clinical decision-making in routine oncology practice. Prospective studies in larger and independent cohorts are required to validate the clinical applicability and generalizability of ACBS-based prognostic models in metastatic colorectal cancer.

## 5. Conclusions

The findings of our study suggest that ACBS and its modified forms, mACBS-1 and mACBS-2, represent practical biomarkers for predicting OS in patients with metastatic colorectal cancer. Derived from routinely available laboratory parameters, ACBS provides a cost-effective and easily accessible tool for clinical risk stratification that can be readily implemented in daily practice. Moreover, identification of patients with higher ACBS-based risk scores may facilitate early recognition of individuals with increased inflammatory and nutritional burden, potentially allowing closer monitoring and supportive interventions. Nevertheless, further prospective studies in larger, independent, and multicenter mCRC cohorts are warranted to validate these findings and to clarify the role of ACBS-based models in routine clinical decision-making.

## Figures and Tables

**Table 1:** Demographic and Clinical Characteristics of Patients.

Variable	Total (*n* = 179)
Age (Mean ± SD/Min-Max)	61.8 ± 12.8 (38–82)
Sex	***n*** (%)
Male	108 (60.3)
Female	71 (39.7)
ECOG	
0–1	166 (92.7)
2–3	13 (7.3)
Stage at initial diagnosis	
Stage II	17 (10.0)
Stage III	46 (27.1)
Stage IV	107 (62.9)
Primary tumor location	
Cecum	7 (3.9)
Right colon	34 (19.0)
Left colon	14 (7.8)
Transverse colon	5 (2.8)
Sigmoid colon	55 (30.7)
Rectum	64 (35.8)
Surgical resection of primary tumor	
Yes	141 (78.8)
No	38 (21.2)
Mutational Status	
RAS mutation	113 (63.1)
RAS wild-type	65 (36.3)
Unknown	1 (0.6)
BRAF V600E mutation	10 (5.6)
BRAF wild-type	75 (41.9)
Unknown	94 (52.5)
Mismatch repair status	
MSI-high	3 (1.7)
MSI-low	85 (47.5)
Unknown	91 (50.8)
Metastatic presentation	
De novo metastatic	115 (64.2)
Recurrence	64 (35.8)
Sites of metastasis	
Liver	118 (65.9)
Lung	70 (39.3)
Bone	23 (12.8)
Brain	3 (1.7)
Other sites	88 (49.2)
First-line systemic treatment	
Chemotherapy + anti-VEGF	133 (74.3)
Chemotherapy + anti-EGFR	46 (25.7)
ACBS	
0	62 (34.6)
1	54 (30.2)
2	47 (26.3)
3	16 (8.9)
mACBS-1	
Favorable	63 (35.2)
Intermediate	100 (55.9)
Poor	16 (8.9)
mACBS-2	
Favorable	117 (65.4)
Poor	62 (34.6)
Best response to treatment	
Complete response	33 (20.2)
Partial response	46 (28.2)
Stable disease	24 (14.7)
Progressive disease	60 (36.8)
Number of treatment lines	
1	53 (29.6)
2	74 (41.3)
3	38 (21.2)
4	12 (6.7)
≥5	2 (1.1)
Death	
No	51 (28.5)
Yes	128 (71.5)
Follow-up duration (months) (Mean ± SD/Min-Max)	34.4 ± 27.2 (1–144)

Data are presented as number (*n*) of patients and percentage (%). Abbreviations: SD, standard deviation; ECOG, Eastern Cooperative Oncology Group; MSI, microsatellite instability; EGFR, epidermal growth factor receptor; ACBS, Aarhus composite biomarker score; mACBS-1, modified Aarhus composite biomarker score-1; mACBS-2, modified Aarhus composite biomarker score-2.

**Table 2:** Univariate Cox regression analysis of overall survival.

Variable		*p*	HR	95% CI	
				Min	Max
Age		0.291	1.007	0.994	1.022
Gender		0.881	1.028	0.719	1.468
Surgery		0.002	0.497	0.319	0.773
Localization	cecum	0.092			
right colon		0.567	1.522	0.361	6.428
left colon		0.670	1.392	0.304	6.374
rectum		0.285	2.163	0.526	8.893
transverse colon		0.590	0.583	0.082	4.147
sigmoid colon		0.769	1.239	0.296	5.182
RAS status	mutant	0.026			
wild		0.566	0.898	0.622	1.296
unknown		0.009	15.107	1.943	117.449
BRAF status	mutant	0.875			
wild		0.915	0.957	0.425	2.154
unknown		0.880	1.061	0.490	2.298
MSI status	high	0.286			
low		0.826	0.853	0.206	3.524
unknown		0.854	1.142	0.279	4.676
De novo metastatic		˂0.001	2.161	1.495	3.123
Site of metastasis	liver	0.002	1.822	1.250	2.657
lung		0.745	1.061	0.745	1.510
bone		0.155	1.438	0.871	2.372
brain		0.306	0.358	0.050	2.564
Total number of treatments					
1		0.829			
2		0.578	0.879	0.558	1.385
3		0.646	0.888	0.536	1.473
4		0.224	0.626	0.294	1.332
5		0.962	0.000	0.000	
ACBS (Ref: normal results)		˂0.001			
1 result abnormal		0.258	1.295	0.827	2.026
2 results abnormal		˂0.001	2.326	1.475	3.670
3 results abnormal		0.001	2.819	1.494	5.319
mACBS-1 (Ref: favorable risk)		0.003			
intermediate risk		0.015	1.619	1.099	2.387
poor risk		0.002	2.776	1.474	5.228
mACBS-2	poor risk	˂0.001	2.105	1.469	3.017

Abbreviations: HR, hazard ratio; CI, confidence interval; ACBS, Aarhus composite biomarker. score; mACBS-1, modified Aarhus composite biomarker score-1; mACBS-2, modified Aarhus composite biomarker score-2.

**Table 3:** Multivariate Cox regression analysis of overall survival.

		*p*	HR	95% CI	
				Min	Max
Surgery		0.600	0.843	0.445	1.598
De novo metastatic		0.015	1.749	1.114	2.744
Site of metastasis	liver	0.090	1.480	0.941	2.328
bone		0.025	1.901	1.083	3.337
ACBS (Ref: normal results)		˂0.001			
1 result abnormal		0.821	1.061	0.633	1.781
2 results abnormal		0.040	1.837	1.027	3.286
3 results abnormal		˂0.001	4.612	2.165	9.824
mACBS-1 (Ref: favorable risk)		0.006			
intermediate risk		0.101	1.473	0.928	2.339
poor risk		0.002	3.356	1.583	7.115
mACBS-2	poor risk	0.002	2.050	1.291	3.256

Abbreviations: HR, hazard ratio; CI, confidence interval; ACBS, Aarhus composite biomarker score; mACBS-1, modified Aarhus composite biomarker score-1; mACBS-2, modified Aarhus composite biomarker score-2.

## Data Availability

The data are not publicly available due to patient privacy and institutional policies; however, they are available from the corresponding author upon reasonable request.

## Supplementary Materials

The following supporting information can be downloaded at: https://www.mdpi.com/article/10.3390/diagnostics16060863/s1, Supplementary Table S1: Distribution of KRAS and BRAF mutations across ACBS subgroups; Supplementary Table S2: Distribution of KRAS and BRAF mutations across mACBS-1 subgroups; Supplementary Table S3: Distribution of KRAS and BRAF mutations across mACBS-2 subgroups; Supplementary Table S4: Distribution of systemic treatment regimens according to ACBS and modified ACBS risk groups; Supplementary Table S5: Prognostic performance of ACBS and modified ACBS risk groups.

## Abbreviations

The following abbreviations are used in this manuscript:

mCRC	Metastatic colorectal cancer
ACBS	Aarhus composite biomarker score
mACBS-1	modified Aarhus composite biomarker score-1
mACBS-2	modified Aarhus composite biomarker score-2
OS	overall survival
CRC	Colorectal cancer
CRP	C-reactive protein
NLR	Neutrophil-to-lymphocyte ratio
LCR	Lymphocyte-C-reactive protein ratio
SII	systemic immune-inflammation index
SIRI	Systemic Inflammation Response Index
GPS	Glasgow Prognostic Score
