# Supplementary material for: Modified Aarhus Composite Biomarker Score as a New Risk-Stratification Tool in Metastatic Colorectal Cancer
Source: Diagnostics (Basel). 2026 Mar 13;16(6):863. doi: 10.3390/diagnostics16060863 (PMC13024963; doi:10.3390/diagnostics16060863)
Supplement: Supplementary file 1 [file diagnostics-16-00863-s001.zip › diagnostics-4158687-supplementary.pdf]

Supplementary Table S1. Distribution of KRAS and BRAF mutations across ACBS subgroups.

ABCS	K-RAS Mutant, n/N (%)	BRAF Mutant, n/N (%)
normal results	40/62 (64.5)	7/62 (11.3)
1 result abnormal	33/54 (61.1)	1/54 (1.9)
2 results abnormal	31/47 (66.0)	2/47 (4.3)
3 results abnormal	9/16 (56.2)	0/16 (0.0)
***p* - value**	0.780	0.380

Abbreviations: ACBS, Aarhus composite biomarker score

Supplementary Table S2. Distribution of KRAS and BRAF mutations across mACBS-1 subgroups.

mACBS-1	K-RAS Mutant, n/N (%)	BRAF Mutant, n/N (%)
favorable risk	41/63 (65.1)	7/63 (11.1)
intermediate risk	63/100 (63.0)	3/100 (3.0)
poor risk	9/16 (56.2)	0/16 (0.0)
***p* - value**	0.884	0.156

Abbreviations: mACBS-1, modified Aarhus composite biomarker score-1

Supplementary Table S3. Distribution of KRAS and BRAF mutations across mACBS-2 subgroups.

mACBS-2	K-RAS Mutant, n/N (%)	BRAF Mutant, n/N (%)
favorable risk	74/117 (63.2)	8/117 (6.8)
poor risk	39/62 (62.9)	2/62 (3.2)
***p* - value**	0.508	0.548

Abbreviations: mACBS-2, modified Aarhus composite biomarker score-2

Supplementary Table S4. Distribution of systemic treatment regimens according to ACBS and modified ACBS risk groups.

	Risk Group	n	Anti-VEGF, n (%)	Anti-EGFR, n (%)	*p*
ACBS	Normal results	62	50 (80.6)	12 (19.4)	0.198
	1 result abnormal	54	37 (68.5)	17 (31.5)	
	2 results abnormal	47	32 (68.1)	15 (31.9)	
	3 results abnormal	16	14 (87.5)	2 (12.5)	
mACBS-1	Favorable	63	51 (81.0)	12 (19.0)	0.082
	Intermediate	100	68 (68.0)	32 (32.0)	
	Poor	16	14 (87.5)	2 (12.5)	
mACBS-2	Favorable	117	87 (74.4)	30 (25.6)	1.000
	Poor	62	46 (74.2)	16 (25.8)	

Abbreviations: ACBS, Aarhus composite biomarker score; mACBS-1, modified Aarhus composite biomarker score-1; mACBS-2, modified Aarhus composite biomarker score-2

Supplementary Table S5. Prognostic performance of ACBS and modified ACBS risk groups.

	**Harrell C-Index**	**AUC (12 Months)**	**AUC (36 Months)**	**AUC 60 Months)**	**Mean AUC**	**AIC**	**Likelihood Ratio Test *p*-Value**
ACBS	0.659	0.818	0.674	0.580	0.690	1083.21	<0.001
mACBS-1	0.620	0.742	0.640	0.543	0.642	1088.18	0.003
mACBS-2	0.632	0.789	0.623	0.578	0.663	1081.97	<0.001

Abbreviations: ACBS, Aarhus composite biomarker score; mACBS-1, modified Aarhus composite biomarker score-1; mACBS-2, modified Aarhus composite biomarker score-2; AUC, area under curve.
